# Chimeras could help in the fight against leptospirosis

**DOI:** 10.7554/eLife.34087

**Published:** 2018-01-19

**Authors:** Jademilson C Santos, Ana Lucia TO Nascimento

**Affiliations:** Laboratório Especial de Desenvolvimento de Vacinas-Centro de BiotecnologiaInstituto ButantanSão PauloBrazil

**Keywords:** LigB structure, mAb accessibility, vaccine design, Other

## Abstract

Understanding the structure of an antigen can guide the design of improved antigen-based vaccines.

**Related research article** Hsieh CL, Ptak CP, Tseng A, Suguiura IMS, McDonough SP, Sritrakul T, Li T, Lin YP, Gillilan RE, Oswald RE, Chang YF. 2017. Extended low-resolution structure of a *Leptospira* antigen offers high bactericidal antibody accessibility amenable to vaccine design. *eLife* **6**:e30051. doi: 10.7554/eLife.30051

Leptospirosis is a disease that affects humans and animals worldwide, with a high prevalence in tropical and subtropical regions. The *Leptospira* bacteria that cause the disease colonize the kidneys of wild and domestic animals, and humans come into contact with these bacteria via the urine of infected animals – primarily rats in urban areas (Bharti et al., 2003). Symptoms of leptospirosis range from a mild influenza-like illness to severe infections that are fatal in over half of cases (Marotto et al., 1999). The lack of an effective vaccine has hampered efforts to prevent and control the disease.

Vaccines contain substances that allow the host immune system to learn how to recognize a particular pathogen. The parts of the pathogen that are recognized by the immune system are known as antigens, and immune molecules called antibodies bind to these antigens as part of the immune response.

Current vaccines against leptospirosis consist of whole inactivated bacterial cells, which induce the host immune system to produce antibodies against lipopolysaccharide molecules in the outer membrane of the bacteria. However, these vaccines only provide short-term immunity against the specific varieties of bacteria that are included (in their inactivated form) in the vaccine (Adler and de la Peña Moctezuma, 2010). Moreover, there are more than 250 serum varieties of *Leptospira*, so developing a vaccine that is effective against all of them with this methodology is unrealistic.

Advances in recombinant DNA techniques, allied to whole-genome sequencing and bioinformatics technologies, have led to a new approach for the identification of vaccine candidates. This approach, known as reverse vaccinology, uses the genome of the pathogen to predict the exact part of an antigen that antibodies interact with (Rappuoli et al., 2014). However, despite these advances, only a few antigens that protect against leptospirosis have been identified. Now, in eLife, Yung-Fu Chang and Robert Oswald of Cornell University and colleagues – including Ching-Lin Hsieh and Christopher Ptak as joint first authors – report a valuable step forward in efforts to develop an effective vaccine against pathogenic *Leptospira* (Hsieh et al., 2017).

LigB is a protein found on the surface of pathogenic forms of *Leptospira* (Matsunaga et al., 2003). It contains a short N-terminal domain (which anchors it to the outer membrane of the bacterium), twelve consecutive immunoglobulin-like domains (called LigB1-12), and a large non-immunoglobulin-like domain at the C-terminal end. The 12 central domains can be divided into a conserved region (LigB1-7) and a more variable region (LigB7-12; Ptak et al., 2014). LigB has been considered the most promising target for an effective vaccine (Conrad et al., 2017), but controversial results suggested only a partial immunization (Yan et al., 2009; Silva et al., 2007) or did not confer sterilizing immunity (protection against infection as well as disease; Evangelista et al., 2017).

Hsieh et al. combined structural biology with immunoreactive assays to determine the region of the LigB protein that most strongly induces an immune response. They used small-angle X-ray scattering to determine the low-resolution structure of the LigB1-12 region by working their way along this region, imaging five of the domains at a time. The final structure demonstrated an extensive surface area that is present across almost all of the 12 domains. This provides a high degree of exposure to the host immune system.

To confirm the capability of the protein to induce a host immune response, Hsieh et al. used two truncated forms of LigB – one that consisted of LigB1-7, and one formed of LigB7-12 – to generate a library of anti-LigB monoclonal antibodies. The bactericidal activity of these antibodies was evaluated by measuring how they interacted with LigB and how well they adhered to the surface of pathogenic *Leptospira*. These interactions were then correlated with the ability of the monoclonal antibodies to kill the bacteria in the presence of innate immune proteins called serum complement. By blocking important domains of LigB, monoclonal antibodies render *Leptospira* susceptible to attack and killing by complement proteins.

Using a technique called nuclear magnetic resonance spectroscopy, Hsieh et al. worked out the structure of the monoclonal antibody domains that have bactericidal activity. These data helped them to build chimera proteins from selected domains that were then used to immunize hamsters against virulent *Leptospira* (Figure 1). One chimera containing just three domains – LigB10-B7-B7 – afforded better protection to hamsters than longer constructs, such as LigB7-12 (which contains six domains).

**Figure 1.**
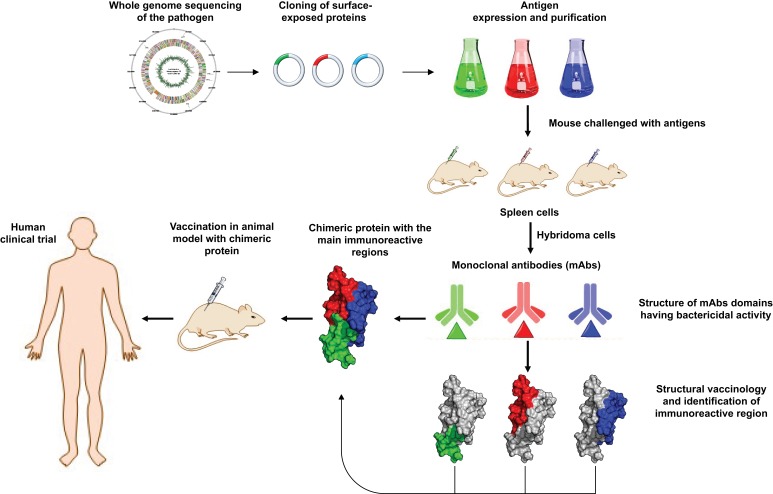
Schematic representation of how structural biology contributes to vaccine design. Sequencing the genome of a pathogen (top left) makes it possible to clone protein-based antigens – the features of the pathogen that are detected by antibodies in the host immune system. When mice are injected with the purified antigens, cells in their spleen produce monoclonal antibodies (mAbs) via cells called hybridomas. By studying the structure of the antibodies, and identifying the regions that interact most strongly with the antigens, researchers can build chimeric proteins from these regions. The effectiveness of the chimera as a vaccine for the pathogen can then be tested in animal models and human clinical trials.

The results of Hsieh et al. reinforce previous work that showed that structural biology represents a powerful tool for structure-based vaccine design. Their findings have significantly advanced our knowledge of LigB and represent an important step toward an improved vaccine against leptospirosis.
